# Correction: Media representations of China’s inclusive education: A corpus-assisted critical discourse analysis

**DOI:** 10.1371/journal.pone.0317019

**Published:** 2024-12-31

**Authors:** Yating Yu, Gaoqiang Lu, Kuen Fung Sin, Zhiying Yu

There is an error in affiliation 3 for author Kuen Fung Sin. The correct affiliation 2 is: EdUHK to The Department of Special Education and Counselling, Faculty of Education and Human Development, The Education University of Hong Kong.

The correct citation is: Yu Y, Lu G, Sin K. F, Yu Z (2024) Media representations of China’s inclusive education: A corpus-assisted critical discourse analysis. PLoS ONE 19(3): e0300123. https://doi.org/10.1371/journal.pone.0300123

## References

[1] Yu Y, Lu G, Sin K. F, Yu Z (2024) Media representations of China’s inclusive education: A corpus-assisted critical discourse analysis. PLoS ONE 19(3): e0300123. doi: 10.1371/journal.pone.0300123 38547159 PMC10977776

