# Supplementary material for: Media representations of China’s inclusive education: A corpus-assisted critical discourse analysis
Source: PLoS One. 2024 Mar 28;19(3):e0300123. doi: 10.1371/journal.pone.0300123 (PMC10977776; doi:10.1371/journal.pone.0300123)
Supplement: Attachment — Submitted filename: Reply to Editor Dec 2023.docx [file pone.0300123.s001.docx]

This manuscript presents a corpus-assisted discourse analytic research on the Chinese government initiative for inclusive education. Using English-language local news channels, the researchers examined 73 news articles for meticulous line-by-line concordance analysis. The researchers endeavoured to review China’s self-image regarding the initiative of inclusive education. Results have named three major categories (i.e. efforts to develop inclusive education, benefits of inclusive education and challenges before inclusive education). Overall, the paper is adequately executed and the analyses performed are appropriate to answer the research questions. The authors are off to a good start, however, this study requires additional exploration of some news articles that is not belong to “party-controlled news media” platforms for more unbiased findings. Otherwise, this methodological issue can limit the results of the study and the discussion.

Moreover, authors are advised to build their results section on the exhibition of the three main categories they have found. And let the discussion be leaded by their research questions compared to the related literature. Ultimately, the study presenting well-promising idea and adding to the current knowledge. Consequently, I would recommend it for publication with relatively major correction.

The research questions seems to be underdeveloped and need more clarification. For instance, what they mean by ‘the linguistic patterns’ in the second question? However, literature review relatively present an overview of the initiative of inclusive education with little coverage of related literature. Readers need to be convinced by your knowledge gap, so firstly presents what others conducted followed by what you will add. However, you argued that your study will unveil the influence of news media on in shaping foreign public perceptions. I do not think you have explored foreign public perceptions to know the level of influence of these media channels on them.

On methods, your selection of the tool Factiva needs to be justified, what is it? Is it a database rolled by the government or a private sector? Have you tested its validity and reliability? Furthermore, the authors noted that the extract data (i.e. 137 articles) was filtered manually, the possibility of errors being included cannot be ruled out. Therefore, it may be more reliable to use the original electronic data for filtrations. Otherwise, manual filtrations can still be an option by some experts consultation. Another eye for such manual process seems inevitable.

There is a need for segregation between results & discussion sections. Authors should try to represent their results at the findings section and leave discussion and/or comparison to the discussion part. For example, your discussion of examples 11, 12 and 13 on pages 23 & 24 should be moved to the discussion part. In addition, the study fails to address how the findings relate to previous research in this area. The authors should rewrite their discussion to reference the related literature, especially recently published work.

Ultimately, the current manuscript requires significant methodological changes for it to be considered for publication. Overall, the topic explored in this paper is engaging, yet the limitations identified should not be ignored.

**Minor corrections**

**Page 9 at the Introduction--->** Try to rephrase the sentence starts with ‘However, due to... ’.

**Page 11 Paragraph 2--->** Mention the findings of Florian and Linklater.

**Page 13 Paragraph 1--->** What do you mean by the number 500 after ConcGram?

**Page 13 Paragraph 1--->** At the bottom, mention the page number for the quote.

**Page 14 Paragraph 2--->** You mentioned the category (others) at Table 2, try to provide some examples for that category.

**Page 18 Paragraph 1--->** The sentence “This echoes previous....” And the one followed should be at the discussion part. Please see my prior comment about differentiating between findings’ explanations and discussion.

**Page 21 Paragraph 1--->** Same remark as the previous comment.

**Page 25 Paragraph 1--->** Sentence starts with “This educational model enables...” until the end seems repetitive and through unnecessary, please omit it.

**Page 26 Paragraph 2--->** Move that to the discussion section.

**Reference 21 --->** “2020; 10” Is the 10 referring to article’s pages number? If yes then mention the last page number.

**Reference 22 --->** Same comment the previous one.

**Reference 31 --->** Do you mean China Daily?

**Reference 35 --->** Make sure that is the correct reference style.

**Reference 37 --->** What “100912” mean?

**Reference 55 --->** Unnecessary bracket usage and revise pages number.
