# Supplementary material for: Media representations of China’s inclusive education: A corpus-assisted critical discourse analysis
Source: PLoS One. 2024 Mar 28;19(3):e0300123. doi: 10.1371/journal.pone.0300123 (PMC10977776; doi:10.1371/journal.pone.0300123)
Supplement: Attachment — Submitted filename: Response to Reviewers.docx [file pone.0300123.s002.docx]

| **Reviewer #1** | **Our response** |
| --- | --- |
| The study addressed an important topic but the manuscript needs to be substantially revised. I suggest that the authors consider addressing the following issues: | Thank you for taking the time to review our manuscript and for your helpful feedback. We appreciate your thoroughness and attention to detail, and we are glad to hear that you found our topic to be important. Your feedback has been invaluable in improving the quality of our work, and we are grateful for your time and effort. We have carefully modified the relevant sections – highlighted in colour – in response to your perceptive comments. |
| (1) It is necessary to strengthen the link between the project's objective and the research questions. Why did the analysis focus on linguistic and discourse analysis? As a reader, I would like to read that the research questions are compellingly justified by the writing. (2) I do not think that the literature review connects to the research questions well. Although readers need some contextual understanding, the authors should really elaborate on the argument for the research questions to be addressed.   It is also necessary for the authors to present any conceptual framework that might have underpinned the analysis. Otherwise, it will be a very descriptive analysis.   (3) Can the authors be explicit about how each research question was addressed in the analysis of data? (4) I do not read a clear alignment between research questions and findings. It is not clear whether both questions were thoroughly answered by the results.   The results are also very descriptive suggesting that there is a need to go beyond the surface level of analysis (e.g. counting).   (5) How do the findings add to the existing body of literature on the topic? This is a problem for this reader as the authors did not contextualize the study with the relevant disciplinary field (e.g., is this a study for readers in special education or public communication?). As a result, I am not sure in what sense the findings are significant new knowledge. The authors should really go back to the introduction and literature review to articulate a clear argument for the study by contextualizing it within a significant disciplinary knowledge gap. (6) Any thoughts on the implications that the study has for readers? Implications for research and practice? | (1) The objective of the project centres on the coverage of China’s state-controlled news media regarding its inclusive education initiatives aimed at international audiences, with the intention of enhancing its national image. Therefore, this study asked questions about how the state-controlled media built its national image by using different discourses. To address these research questions, we have employed a corpus-assisted critical discourse approach. Firstly, this linguistic approach enables us to delve into a substantial volume of data, thereby mitigating researcher bias. Secondly, it allows us to discern the linguistic and discursive techniques employed by the state-controlled media to bolster its national image. Following the formulation of the research questions, we have elucidated the connections between the various components. Please read, “We employed corpus-assisted critical discourse analysis to address these two questions...the state-run media to construct persuasive discourses and uncover power dynamics.”. (2) We apologise that our literature review was not sufficiently clear in presenting the argument for the research questions and identifying our research gap, which is to examine the impact of state-controlled news media on shaping foreign public perceptions of Intellectual IE in China. In order to address this research gap and provide a more detailed explanation of the argument for the research questions, we have conducted a thorough review of various studies that investigate how the Chinese government utilised its state-controlled news media to influence how China is perceived by foreign audiences, particularly in the context of the COVID-19 pandemic. Please read, “2.1. National image and state-run news media”.   In response to your comment, we acknowledge that our analysis may not have provided a clear enough conceptual framework. Therefore, we have included an additional sub-section titled “corpus-assisted critical discourse analysis” in the literature review. This section presents the conceptual framework utilised in our study. Furthermore, we have examined previous studies employing a similar approach and critiqued them to avoid being too “descriptive” in Reviewer #1’s words. Although these reviewed studies have contributed valuable insights into the application of the corpus-assisted CDA approach to analyse media data, there is still room for our study to provide complementary findings. Please refer to section “2.2. Corpus-assisted critical discourse analysis” for further details. To avoid redundant information in the literature review, we have provided specific details of the corpus-assisted discourse approach, particularly in relation to semantic prosody, in the method section.   (3) We have explicitly addressed how we answered each research question in the “Method” section. Please read, “To answer the first research question. To address the second research question…”. (4) In order to ensure a precise correspondence between the research questions and our findings, we have addressed each question right at the start of the “findings” section. Please read, “From the 520 co-occurring results, they can be broadly categorised into four groups…”.   In order to steer clear of excessive descriptiveness and quantification, which are known weaknesses of corpus linguistics, we have opted to adopt the corpus-assisted CDA approach. This approach allows us to delve deeper into language patterns, going beyond the superficial level, as we highlighted in our literature review. As a result, we have reexamined our findings to incorporate critical discourse analysis.   (5) We apologise for the confusion. We have revised the introduction, literature review, and discussion to refine our focus. Our study aims to contribute fresh insights to the field of communication. (6) We have written the implications that the study has for readers in the section “6. Conclusion”. Regarding implications for research, please read, “We offer a model for conducting corpus-assisted CDA...”. As for practice, please read, “Certain issues deserve attention...”. |
|  | Once again, thank you for your insightful and helpful comments on our paper. We sincerely appreciate the time and effort you spent assisting us in improving the quality of our paper. We hope the changes live up to your expectations and appropriately resolve any issues you had with the paper in the past. We are incredibly grateful for your helpful advice and assistance. |

| **Reviewer #2** | **Our response** |
| --- | --- |
| This manuscript presents a corpus-assisted discourse analytic research on the Chinese government initiative for inclusive education. Using English-language local news channels, the researchers examined 73 news articles for meticulous line-by-line concordance analysis. The researchers endeavoured to review China’s self-image regarding the initiative of inclusive education. Results have named three major categories (i.e. efforts to develop inclusive education, benefits of inclusive education and challenges before inclusive education). Overall, the paper is adequately executed and the analyses performed are appropriate to answer the research questions. | We would like to thank you for your nice remarks and for recognising the work that we have done. We appreciate the fact that you think the paper is adequately executed and the analyses performed are appropriate to answer the research questions. We are very impressed by your detailed, constructive, and useful comments. We have carefully changed the pertinent sections – which are highlighted in colour – in response to your insightful remarks. |
| (1) The authors are off to a good start, however, this study requires additional exploration of some news articles that is not belong to “party-controlled news media” platforms for more unbiased findings. Otherwise, this methodological issue can limit the results of the study and the discussion. (2) Moreover, authors are advised to build their results section on the exhibition of the three main categories they have found. And let the discussion be leaded by their research questions compared to the related literature. Ultimately, the study presenting well-promising idea and adding to the current knowledge. Consequently, I would recommend it for publication with relatively major correction. (3) The research questions seem to be underdeveloped and need more clarification. For instance, what they mean by ‘the linguistic patterns’ in the second question? However, literature review relatively present an overview of the initiative of inclusive education with little coverage of related literature. Readers need to be convinced by your knowledge gap, so firstly presents what others conducted followed by what you will add. However, you argued that your study will unveil the influence of news media on in shaping foreign public perceptions. I do not think you have explored foreign public perceptions to know the level of influence of these media channels on them. (4) On methods, your selection of the tool Factiva needs to be justified, what is it? Is it a database rolled by the government or a private sector? Have you tested its validity and reliability? Furthermore, the authors noted that the extract data (i.e. 137 articles) was filtered manually, the possibility of errors being included cannot be ruled out. Therefore, it may be more reliable to use the original electronic data for filtrations. Otherwise, manual filtrations can still be an option by some experts consultation. Another eye for such manual process seems inevitable. (5) There is a need for segregation between results & discussion sections. Authors should try to represent their results at the findings section and leave discussion and/or comparison to the discussion part. For example, your discussion of examples 11, 12 and 13 on pages 23 & 24 should be moved to the discussion part. In addition, the study fails to address how the findings relate to previous research in this area. The authors should rewrite their discussion to reference the related literature, especially recently published work. Ultimately, the current manuscript requires significant methodological changes for it to be considered for publication. Overall, the topic explored in this paper is engaging, yet the limitations identified should not be ignored, | (1) Our objective is to examine China’s self-constructed image in the context of its efforts towards inclusive education. Hence, we sought to explore how the Chinese Government portrays its image with regard to inclusive education. This led us to investigate state-controlled news media, which serve as a platform for expressing the government’s viewpoint. Nevertheless, we are aware of the constraints inherent in our study. In light of this, we have revised our research and proposed that future studies include an examination of non-state-controlled news media, enabling a comparison with our conclusions. Please read, “A limitation of this study lies in...”. (2) We have revised our results and discussion. The results centre on the three primary categories, and we have compared our findings with the relevant literature in the discussion section, which is guided by our research questions. (3) We have revised and clarified both research questions. The project’s objective centres on China’s state-controlled news media coverage regarding its inclusive education initiatives aimed at international audiences, with the goal of enhancing its national image through the use of discourses and the employed discursive strategies to construct these discourses.   We apologise that our literature review was not sufficiently clear in presenting the argument for the research questions and identifying our research gap, which is to examine the impact of state-controlled news media on shaping foreign public perceptions of Intellectual IE in China. In order to address this research gap and provide a more detailed explanation of the argument for the research questions, we have conducted a thorough review of various studies that investigate how the Chinese government utilised its state-controlled news media to influence how China is perceived by foreign audiences, particularly in the context of the COVID-19 pandemic. Please read, “2.1. National image and state-run news media” and “2.2. Corpus-assisted critical discourse analysis”.   (4) Factiva is a commercial news database owned by Dow Jones. In terms of its validity and reliability, it has been extensively utilised by researchers from diverse fields.   To mitigate the potential for errors and ensure an additional layer of scrutiny, two authors independently conducted manual screening to assess the relevance of all news articles. They also performed an inter-rater reliability assessment. The high level of agreement, as indicated by a Kappa Coefficient test result of 0.98, demonstrates strong consistency.  Please read, “Factiva is a commercial news database owned by Dow Jones, which has been widely utilised by researchers across diverse disciplines [27,28]…Furthermore, two authors independently conducted a meticulous assessment to evaluate the pertinence of all the news articles. They also carried out an inter-rater reliability analysis. The outcome of the Kappa Coefficient test, with a value of 0.98, suggests a substantial level of concurrence between the authors [35].”   (5) We have presented our findings in the results section and addressed the discussion and/or comparison in the discussion section. Furthermore, we have revised our discussion to incorporate references to the relevant literature, particularly recently published work. The discussion of examples 11, 12, and 13, originally located on pages 23 and 24, has been relocated to the discussion section. They are underlined. |
| Minor corrections   (6) Page 9 at the Introduction---> Try to rephrase the sentence starts with ‘However, due to... ’. (7) Page 11 Paragraph 2---> Mention the findings of Florian and Linklater. (8) Page 13 Paragraph 1---> What do you mean by the number 500 after ConcGram? (9) Page 13 Paragraph 1---> At the bottom, mention the page number for the quote. (10) Page 14 Paragraph 2---> You mentioned the category (others) at Table 2, try to provide some examples for that category. (11) Page 18 Paragraph 1---> The sentence “This echoes previous....” And the one followed should be at the discussion part. Please read my prior comment about differentiating between findings’ explanations and discussion. (12) Page 21 Paragraph 1---> Same remark as the previous comment. (13) Page 25 Paragraph 1---> Sentence starts with “This educational model enables...” until the end seems repetitive and unnecessary, please omit it. (14) Page 26 Paragraph 2---> Move that to the discussion section. Reference 21 ---> “2020; 10” Is the 10 referring to article’s pages number? If yes then mention the last page number. (15) Reference 22 ---> Same comment the previous one. (16) Reference 31 ---> Do you mean China Daily? (17) Reference 35 ---> Make sure that is the correct reference style. (18) Reference 37 ---> What “100912” mean? (19) Reference 55 ---> Unnecessary bracket usage and revise pages number. | (6) We have rephrased this sentence. Please read, “However, owing to the comparatively later introduction of IE…”. (7) We have mentioned the findings of Florian and Linklater. Please read, “They presented the results of a new initial teacher education course…”. (8) We deleted the number 500. (9) We utilised Mendeley to enter all the references according to the journal’s specifications: the “Vancouver” style. Within our Mendeley software, we have entered the page numbers (p. 65 and p. 157) for the quotes “a feature which extends over more than one unit in a linear string” and “a consistent aura of meaning with which a word is imbued by its collocates”. Nevertheless, it appears that this referencing style does not display page numbers. (10) We have provided some examples for that category (others). Please read, “Parents of disabled students have high hopes...”. (11) We have put the sentences in the discussion section. They are underlined. (12) We have moved some sentences in this paragraph to the discussion section. They are underlined. (13) We have removed the sentences. (14) We have moved this paragraph to the discussion section. They are underlined.   We used Mendeley to input all the references based on the journal’s requirement: the “Vancouver” style. Ten is the Volume. If it is APA, it would be:  Ma, K., & Xiao, Y. (2020). Construction of Huawei’s image in news reports: Take China Daily and The New York Times for example. *International Journal of English Linguistics*, 10 (4), 1–20.   (15) We used Mendeley to input all the references based on the journal’s requirement: the “Vancouver” style. Eleven is the Volume. If it is APA, it would be:   Chen, F. (2021). A study on publicity-orientated translation based on three-dimensional transformation—Taking Twitter of Xinhua News Agency as an example. *Education Research Frontier*, 11(1), 1–5.   (16) We used Mendeley to input all the references based on the journal’s requirement: the “Vancouver” style. It uses “Glob Times” to represent *Global Times* and “China Dly” for *China Daily*, etc. The newspaper names were all correctly input into Mendeley. We have published several papers in *PloS ONE*, and it was not a problem. Thank you for your notice and careless work. (17) The news article entitled “Meeting the Needs of All Pupils” was removed from *Shanghai Daily* but retained in Factiva. In accordance with your suggestion, we have included *Shanghai Daily* as the publication instead of Factiva at the end. (18) It should be the issue number. If it is APA, it would be:   Vantieghem, W., Roose, I., Gheyssens, E., Griful-Freixenet, J., Keppens, K., Vanderlinde, R., Struyven, K., & Van Avermaet, P. (2020). Professional vision of inclusive classrooms: A validation of teachers’ reasoning on differentiated instruction and teacher-student interactions. *Studies in Educational Evaluation*, 67(100912), 1–12.   (19) The bracket signifies the translation of the original Chinese title. We wrote “11-19” in Mendeley. It uses “11-9” to signify “11-19”. It omitted the number “1” in the number “19”.   If it is APA, it would be:  Zhang, M., & Wang, N. (2019). Gaige kaifang sishi nian lai woguo teshu jiaoyu zhengce yanjin yu luoji. [The evolution and logic of special education policies in China over the past forty years]. *ZhongGuo Teshu Jiaoyu [Chinese Journal of Special Education]*, 3, 11–19.  We appreciate your insightful feedback, and we hope we have revised our research to address the points you brought up. These changes hopefully have enhanced the scope and depth of our analysis, improving the study’s overall scholarly contribution. Again, thank you for your helpful suggestions and for pointing us in the direction of a deeper investigation into the RD discourse in a legal and healthcare context in Hong Kong. |

| **Editor/s** | **Our response** |
| --- | --- |
| Dear Dr. Yu,  Thank you for submitting your manuscript to PLOS ONE. After careful consideration, we feel that it has merit but does not fully meet PLOS ONE’s publication criteria as it currently stands. Therefore, we invite you to submit a revised version of the manuscript that addresses the points raised during the review process.  Please submit your revised manuscript by Feb 16 2024 11:59PM. If you will need more time than this to complete your revisions, please reply to this message or contact the journal office at [plosone@plos.org](mailto:plosone@plos.org). When you're ready to submit your revision, log on to … and select the 'Submissions Needing Revision' folder to locate your manuscript file.  Please include the following items when submitting your revised manuscript:   - A rebuttal letter that responds to each point raised by the academic editor and reviewer(s). You should upload this letter as a separate file labeled 'Response to Reviewers'. - A marked-up copy of your manuscript that highlights changes made to the original version. You should upload this as a separate file labeled 'Revised Manuscript with Track Changes'. - An unmarked version of your revised paper without tracked changes. You should upload this as a separate file labeled 'Manuscript'.   If you would like to make changes to your financial disclosure, please include your updated statement in your cover letter. Guidelines for resubmitting your figure files are available below the reviewer comments at the end of this letter.  If applicable, we recommend that you deposit your laboratory protocols in protocols.io to enhance the reproducibility of your results. Protocols.io assigns your protocol its own identifier (DOI) so that it can be cited independently in the future. For instructions read: …. Additionally, PLOS ONE offers an option for publishing peer-reviewed Lab Protocol articles, which describe protocols hosted on protocols.io. Read more information on sharing protocols at …  We look forward to receiving your revised manuscript.  Kind regards,  Chao Gu Academic Editor PLOS ONE  Journal Requirements:  When submitting your revision, we need you to address these additional requirements.  1. Please ensure that your manuscript meets PLOS ONE's style requirements, including those for file naming. The PLOS ONE style templates can be found at  …  2. Thank you for stating the following in your Competing Interests section: “NO authors have competing interests”  Please complete your Competing Interests on the online submission form to state any Competing Interests. If you have no competing interests, please state “The authors have declared that no competing interests exist.”, as detailed online in our guide for authors at...  This information should be included in your cover letter; we will change the online submission form on your behalf.  3. We note that you have indicated that there are restrictions to data sharing for this study. PLOS only allows data to be available upon request if there are legal or ethical restrictions on sharing data publicly. For more information on unacceptable data access restrictions, please read http://journals.plos.org/plosone/s/data-availability#loc-unacceptable-data-access-restrictions.  Before we proceed with your manuscript, please address the following prompts:  a) If there are ethical or legal restrictions on sharing a de-identified data set, please explain them in detail (e.g., data contain potentially identifying or sensitive patient information, data are owned by a third-party organization, etc.) and who has imposed them (e.g., a Research Ethics Committee or Institutional Review Board, etc.). Please also provide contact information for a data access committee, ethics committee, or other institutional body to which data requests may be sent.  b) If there are no restrictions, please upload the minimal anonymized data set necessary to replicate your study findings to a stable, public repository and provide us with the relevant URLs, DOIs, or accession numbers. For a list of recommended repositories, please read... You also have the option of uploading the data as Supporting Information files, but we would recommend depositing data directly to a data repository if possible.  We will update your Data Availability statement on your behalf to reflect the information you provide.  4. When completing the data availability statement of the submission form, you indicated that you will make your data available on acceptance. We strongly recommend all authors decide on a data sharing plan before acceptance, as the process can be lengthy and hold up publication timelines. Please note that, though access restrictions are acceptable now, your entire data will need to be made freely accessible if your manuscript is accepted for publication. This policy applies to all data except where public deposition would breach compliance with the protocol approved by your research ethics board. If you are unable to adhere to our open data policy, please kindly revise your statement to explain your reasoning and we will read the editor's input on an exemption. Please be assured that, once you have provided your new statement, the assessment of your exemption will not hold up the peer review process. | Dear Editor/s,  Thank you for giving us a chance to publish in your reputable journal. We have carefully reviewed your additional requirements and addressed them as follows:  1. We have ensured that our manuscript adheres to PLOS ONE’s style requirements, including those for file naming.  2. We apologise for the oversight in our Data Availability statement regarding the minimal data set underlying our results. We understand the importance of providing access to the necessary data for replicating the study findings. All the data is available from Factiva <https://professional.dowjones.com/factiva/>. Users need to subscribe to Factiva with an amount of subscription fee in order to use it. We have explained how others can access these datasets from the third-party website we accessed in our notes in our revised manuscript. Please read, “Factiva is available at https://professional.dowjones.com/factiva/. Users need to subscribe…”. Upon resubmitting the revised manuscript, we have uploaded the study’s minimal underlying data set to a stable, public repository (https://osf.io/sk5ne/). We have also included the relevant URLs in our revised manuscript. Please read, “The minimal underlying data are publicly available from the OSF repository https://osf.io/sk5ne/” in our notes.  We have also clarified in detail how interested researchers can replicate our study findings in their entirety by directly obtaining the data from the third party, Factiva, and following the information outlined in our Methods section. Please read, “The search format employed for data retrieval was ‘inclusive education*’…”.  Once again, we would like to express our gratitude for your valuable feedback and guidance throughout the submission process.  Best regards,  Yating Yu, Gaoqiang Lu, Sin Kuen Fung, Zhiying Yu |
